# Association of Preadmission Statin Use and Mortality in Critically Ill Patients: A Meta-Analysis of Cohort Studies

**DOI:** 10.3389/fmed.2021.656694

**Published:** 2021-05-28

**Authors:** Shao-shuo Yu, Jian Jin, Ren-qi Yao, Bo-li Wang, Lun-yang Hu, Guo-sheng Wu, Yu Sun

**Affiliations:** ^1^Department of Burn Surgery, Changhai Hospital, The Naval University, Shanghai, China; ^2^Department of Burn and Plastic Surgery, 903rd Hospital of the Chinese People's Liberation Army (PLA), Hangzhou, China

**Keywords:** preadmission use, statin, mortality, critical illness, meta-analysis, propensity score matching

## Abstract

**Background:** A large number of studies have been conducted to determine whether there is an association between preadmission statin use and improvement in outcomes following critical illness, but the conclusions are quite inconsistent. Therefore, this meta-analysis aims to include the present relevant PSM researches to examine the association of preadmission use of statins with the mortality of critically ill patients.

**Methods:** The PubMed, Web of Science, Embase electronic databases, and printed resources were searched for English articles published before March 6, 2020 on the association between preadmission statin use and mortality in critically ill patients. The included articles were analyzed in RevMan 5.3. The Newcastle-Ottawa Scale (NOS) was used to conduct quality evaluation, and random/fixed effects modeling was used to calculate the pooled ORs and 95% CIs. We also conducted subgroup analysis by outcome indicators (30-, 90-day, hospital mortality).

**Results:** All six PSM observational studies were assessed as having a low risk of bias according to the NOS. For primary outcome—overall mortality, the pooled OR (preadmission statins use vs. no use) across the six included studies was 0.86 (95% CI, 0.76–0.97; *P* = 0.02). For secondary outcome—use of mechanical ventilation, the pooled OR was 0.94 (95% CI, 0.91–0.97; *P* = 0.0005). The corresponding pooled ORs were 0.67 (95% CI, 0.43–1.05; *P* = 0.08), 0.91 (95% CI, 0.83–1.01; *P* = 0.07), and 0.86 (95% CI, 0.83–0.89; *P* < 0.00001) for 30-, 90-day, and hospital mortality, respectively.

**Conclusions:** Preadmission statin use is associated with beneficial outcomes in critical ill patients, indicating a lower short-term mortality, less use of mechanical ventilation, and an improvement in hospital survival. Further high-quality original studies or more scientific methods are needed to draw a definitive conclusion.

## Introduction

Statins, which inhibit 3-hydroxy-3-methylglutaryl coenzyme A (HMG-CoA) reductase, are a typical class of medications commonly used for lowering cholesterol levels. There has been substantial evidence proving that statins can play important roles in preventing cardiovascular events and improving patients' survival (1–4). Apart from this well-known therapeutic effect, statins may also have other effects which are lipid-independent. These effects, generally referred to as pleiotropic properties, include anti-inflammatory actions, attenuation of coagulation activation, and immunomodulation (5, 6).

Critically ill patients, such as those who suffer from sepsis, end-stage cardiopulmonary diseases, or severe traumatic injuries, usually have a high prevalence of intensive care unit (ICU) admission and high risk for progression to adverse complications, leading to life-threatening outcomes. Although the exact underlying physiopathological mechanism of various critical illness remains unclear, a large majority of ICU patients suffer from the systemic inflammatory response syndrome (7). Considering the anti-inflammatory property, statins may act as a novel treatment for these critically ill patients in attempt to lower the mortality rate.

Actually, a large number of studies have been conducted to examine whether there is an association between statin use and outcomes following critical illness (8–15), but the conclusions are conflicting as study design varies. Observational studies tend to report beneficial effects with decreased morbidity and mortality of statins on critical illness (16–20), while randomized controlled trials (RCTs) are more likely to demonstrate unfavorable results. This divergence could be partly attributed to the small number and sample size of the available RCTs, which impacts the credibility of the results to some extent. Besides, the pharmacokinetic properties of statins show that no fewer than 2 weeks are needed for the medication to take effect (21–23), indicating the timing of statins therapy is crucial. In fact, most observational studies were conducted on statin users who took statins habitually, while most of the trials were conducted on *de novo* statin users. Considering of the properties of RCT, it is difficult to be applied to the exploration of the efficacy of preadmission administration of statins on patients, so most of the present researches have been prospective or retrospective observational studies with inevitable limitations based on the study type. Previous relevant meta-analyses do provide some indicative results but the inclusion criteria are not very strict and newly published studies should be supplemented to update the conclusion. It is important to include more recent researches with stricter standards into the meta-analysis to further assess if preadmission statin therapy has favorable effect on the outcomes of critical illness. Propensity score matching (PSM), a robust statistical method to balance multiple baseline differences between the investigational groups, has its own advantages in controlling confounding bias. Therefore, this meta-analysis aims to include the present relevant PSM researches to examine the association of preadmission use of statins with the mortality of critically ill patients.

## Materials and Methods

**Figure 1:**
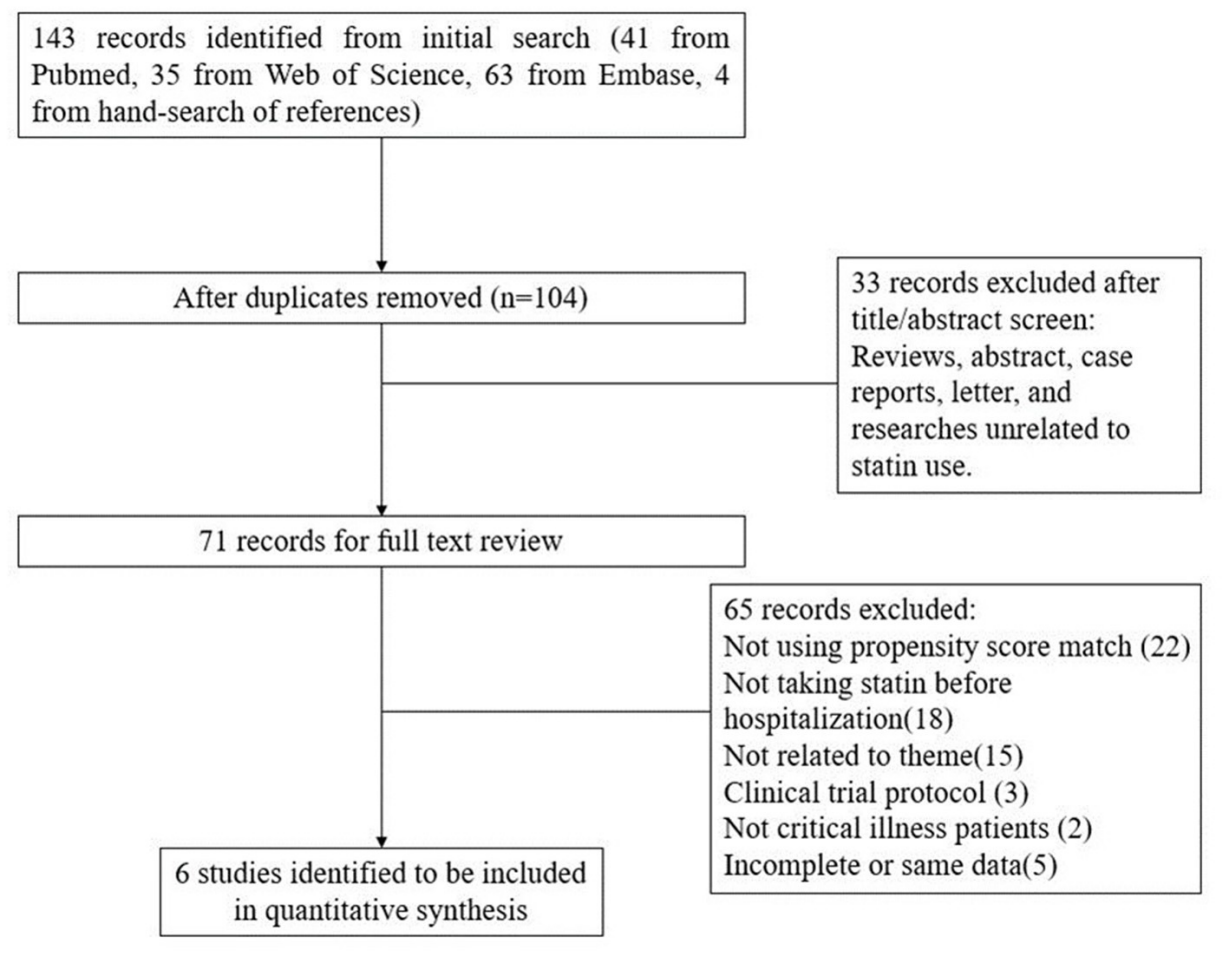
Flowchart for selection process.

This meta-analysis was performed based on the published recommendations and checklist of the Preferred Reporting Items for Systematic Reviews and Meta-Analyses (PRISMA) statement (see Figure 1).

### Search Strategy

We searched the PubMed, Web of Science, and Embase databases for English language articles published from the inception of the database to March 6, 2020, using the following keywords: “statin”, “statins”, “mevastatin”, “simvastatin”, “lovastatin”, “fluvastatin”, “rosuvastatin”, “cerivastatin”, “pravastatin”, “atorvastatin”, “hydroxymethylglutaryl-CoA Reductase Inhibitors”, “critical care”, “critical illness”, “intensive care unit”, “critically ill”, ICU, mortality, death, and propensity. Hand-search resources of the same criteria were also taken into consideration. The flow chart of the study is presented in Figure 1.

### Inclusion Criteria

We included original studies only if they met the following inclusion criteria: (1) Study design: studies analyzed by PSM; (2) Study population: critically ill adult patients (>18 years of age), who were defined as patients admitted to an ICU. When this information was unclear, we considered a mortality rate higher than 5% (hospital mortality or, if this was not reported, ICU mortality, 30-, 60-, 90-day mortality, or long-term mortality) in the control group to be consistent with critical illness; (3) Intervention: critically ill patients who had preadmission statin use vs. those who didn't (control); (4) Study outcomes: mortality should be the primary outcome of included studies, with the rate of mechanical ventilation use could be among the secondary outcomes. We excluded studies that didn't provide clear definition of preadmission intake of statin or had no outcome data.

### Selection of Studies and Data Extraction

In this Meta-analysis, we included observational studies analyzed by PSM that focused on preadmission statin use and critical illness. All the included studies were selected in compliance with the former inclusion criteria by two researchers who worked independently and extracted relevant data, including the name of the first author, publication year, the location of the research, study design, multi/single center, sample demographics (i.e., age, gender), number of patients, and outcome indicators (30-, 60-, 90-, in-hospital, ICU mortality, and mechanical ventilation). Any disagreement was resolved by discussion with a third reviewer for consensus.

### Statistical Analysis

All the quantitative data analyses were conducted in RevMan 5.3. The primary outcome was mortality of critically ill patients with preadmission statin use or not. For those studies that reported multiple types of mortality, the worst indicator of each study would be selected for the pooled effect analysis. Based on this principle, the cardiovascular 90-day mortality from the Oh study, the hospital mortality from the Lokhandwala study, the mid-term mortality (overall all-cause mortality after the mean follow-up period of 33 ± 23 months) from the Vaduganathan study, the 90-day mortalities from the Lee study and the Wiewel study, as well as the 1-year mortality from the Ou study were selected for the pooled overall mortality analysis. The use of mechanical ventilation during hospitalization was also included in the analysis as the secondary outcome. We used *I*^2^ statistic to test the heterogeneity between studies, and an *I*^2^ value of 0, 25, 50, 75% meant no, low, moderate, and high heterogeneity, respectively (24). A fixed or random effects model was used separately when the heterogeneity was low or high, and the pooled OR as well as 95% CI of each outcome were calculated by weighing ORs of each individual study. We also conducted subgroup analysis by outcome indicators (30-, 90-day, hospital mortality). In order to evaluate the appropriateness of eligibility criteria and the stability of included studies, we excluded a single study each time based on the heterogeneity outcome, re-calculated the pooled ORs, and compared them with the original ones. The potential publication bias was assessed with funnel plot (25), which was judged according to the degree of asymmetry of the graph.

## Results

### Study Selection

The initial search yielded 143 articles and 104 remained after duplicates were removed. After title/abstract screening, 71 records were considered potential eligible records relevant to our study object. After full text reviewing, six PSM studies that reported outcomes data of preadmission statin therapy for critical illness were finally identified to be included in this meta-analysis for quantitative synthesis (26–31) (Figure 1).

### Study Characteristics

All the included articles were observational PSM cohort studies which examined the association of preadmission use of statins with outcomes of critically ill patients. Baseline parameters of all the included were well-balanced after PSM. The sample size of these studies ranged from 270 to 55,584 patients with an average age of 56 ± 8 to 70.2 ± 12.3 years. According to the study location, one was conducted in Korea (28), one in Netherlands (30), 2 in USA (27, 31), and the other two in Taiwan (26, 29). According to the types of diseases, three focused on sepsis (26, 29, 30), one on traumatic brain injury (31), one on isolated valve surgery (27), and one on mixed diseases (patients admitted to an ICU) (28). Based on the types of outcomes, three articles reported 30-day mortality (27, 29, 30), three reported 90-day mortality (28–30), and three reported hospital mortality (26, 30, 31), respectively. Among them, four studies also reported the use of mechanical ventilation as the secondary outcome (26, 27, 29, 30). Baseline information about the analyzed studies is presented in Table 1.

**Table 1:** Characteristics of included studies.

**Study**	**Study location**	**Design**	**Multi/Single center**	**preadmission statin use**			**Non-preadmission statin us**			**Outcome**
				**Number**	**Age**	**Male**	**Number**	**Age**	**Male**	
Kyu Oh, 2019 (28)	Korea	Retrospective cohort	Single	5,354	67 ± 12	3,185	7,758	67 ± 14	4,494	90-day mortality
Lokhandwala, 2020 (31)	USA	Observational study	Single	90	56 ± 8	64	180	55 ± 7	126	Hospital mortality
Wiewel, 2018 (30)	Netherlands	Prospective observational study	Single	194	66.7 ± 10.5	123	194	65.8 ± 13.2	121	30-day mortality 60-day mortality 90-day mortality ICU mortality Hospital mortality
Lee, 2017 (29)	Taiwan	Retrospective cohort study	National Database (Multi)	3,325	70.2 ± 12.3	1,605	3,325	70.7 ± 12.1	1,642	30-day mortality 90-day mortality Mechanical ventilation
Ou, 2014 (26)	Taiwan	Retrospective cohort study	National Database (Multi)	27,792	69.1 ± 11.8	11,820	27,792	69.1 ± 11.8	11,820	Hospital mortality One-year mortality Mechanical ventilation
Vaduganathan, 2012 (27)	USA	Retrospective cohort	Single	381	65.3 ± 12.6	218	381	65.1 ± 13.4	223	30-day mortality 90-day mortality ICU mortality Hospital mortality Mechanical ventilation

*ICU, intensive care unit*.

### Risk of Bias Assessment

The risk of bias was assessed by using the Newcastle-Ottawa quality assessment scale (NOS) (32) for all the included observational cohort studies. Based on the subject selection, the comparability between two groups and the reliability of clinical outcomes, the maximum score of NOS is 9. Score 8–9 is considered as excellent quality, 6–7 as good, 5, or below as fair. According to NOS, the six included studies were rated as excellent or good quality (≥7), which indicated a low risk of bias. Details of the risk of bias assessment of all the analyzed studies are presented in Table 2.

**Table 2:** The Newcastle-Ottawa quality assessment scale of included studies.

**Studies**	**Selection**			**Comparability**			**Assessment of outcome**			**Total score**
	**Representativeness of exposure arm(s)**	**Selection of the comparative arm(s)**	**Origin of exposure source**	**Demonstration that outcome of interest was not present at start of study**	**Studies controlling the most important factors**	**Studies controlling the other main factors**	**Assessment of outcome with independency**	**Adequacy of follow-up length**	**Lost to follow-up acceptable**	
Tak Kyu Oh 2019 (28)	^*^	^*^	^*^	^*^	^*^	**-**	^*^	^*^	^*^	**8**
Lokhandwala 2019 (31)	^*^	^*^	^*^	^*^	^*^	**-**	^*^	^*^	^*^	**8**
Wiewel 2018 (30)	^*^	^*^	**-**	^*^	^*^	**-**	^*^	^*^	^*^	**7**
Lee 2017 (29)	^*^	^*^	^*^	^*^	^*^	^*^	^*^	^*^	^*^	**9**
Ou 2014 (26)	^*^	**-**	^*^	^*^	^*^	^*^	^*^	^*^	**-**	**7**
Vaduganathan 2012 (27)	^*^	**-**	^*^	^*^	^*^	^*^	^*^	^*^	^*^	**8**

* *, score 1; **-**, score 0*.

### Effects of Preadmission Use of Statins on Outcomes

**Figure 2:**
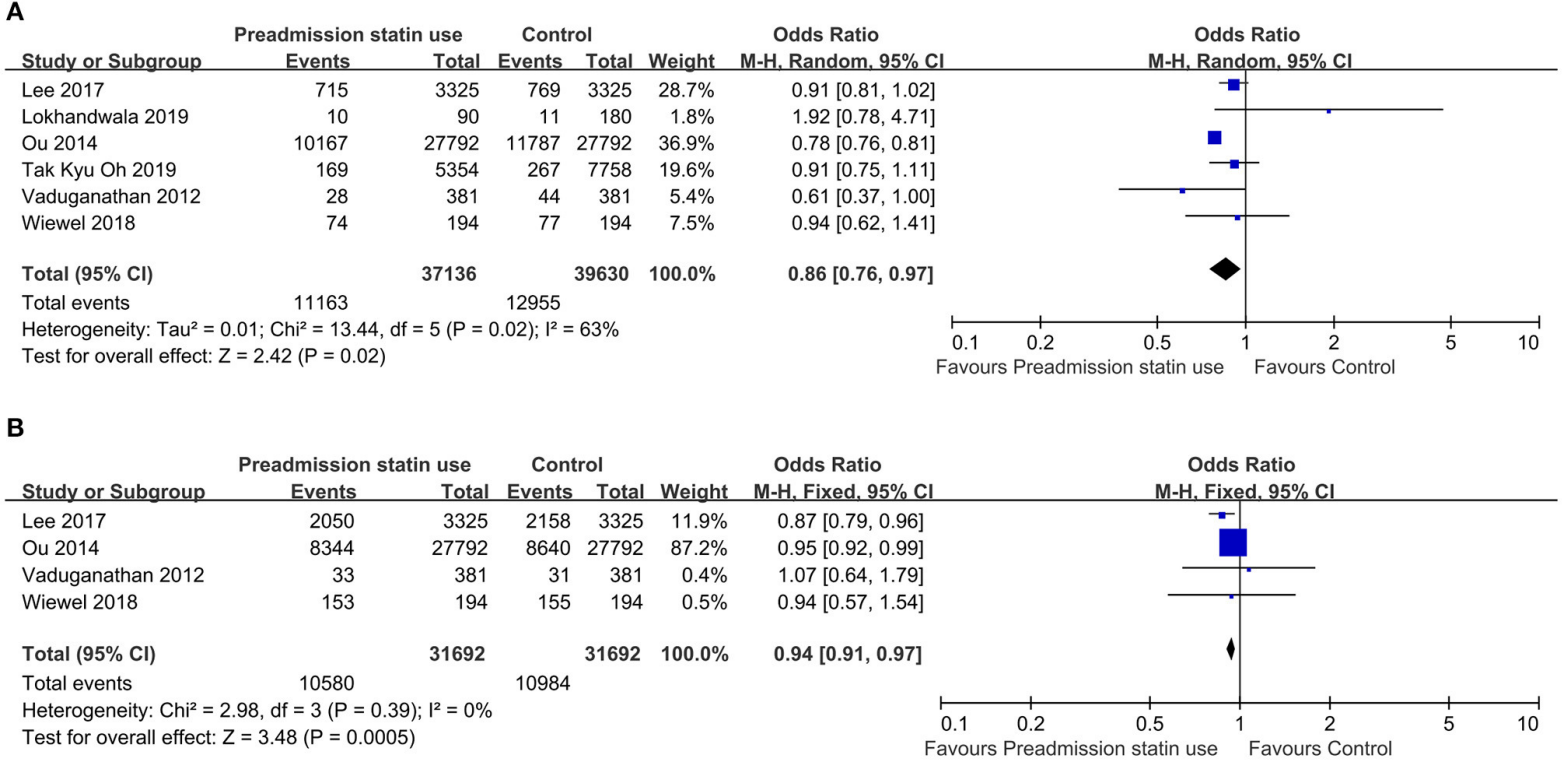
**(A)** Forest plot of overall mortality comparing preadmission statin use to no use in critically ill patients. **(B)** Forest plot of mechanical ventilation comparing preadmission statin use to no use in critically ill patients. CI, confidence interval.

**Figure 3:**
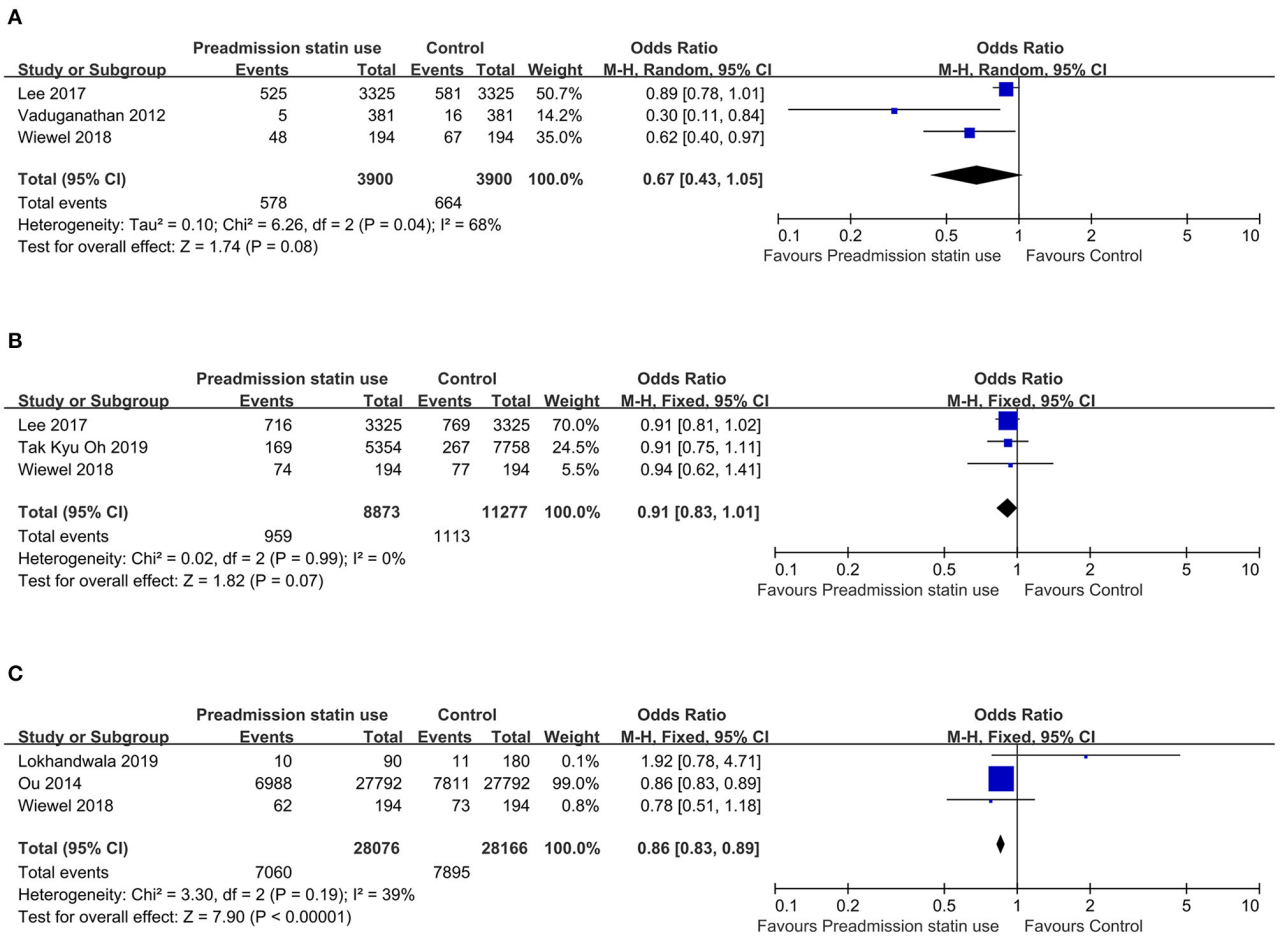
**(A)** Forest plot of 30-day mortality comparing preadmission statin use to no use in critically ill patients. **(B)** Forest plot of 90-day mortality comparing preadmission statin use to no use in critically ill patients. **(C)** Forest plot of hospital mortality comparing preadmission statin use to no use in critically ill patients. CI, confidence interval.

There was substantial heterogeneity across the six studies (*I*^2^ value, 63%), thus a random effect model was used for meta-analysis. For the primary outcome—overall mortality, the pooled OR was 0.86 (95% CI, 0.76–0.97; *P* = 0.02), suggesting that preadmission statin users had significantly lower mortality among critically ill patients compared with non-users (Figure 2A). As there were four included studies concerning the association of preadmission intake of statin with the use of mechanical ventilation, which could also be an indicator to reflect disease progression, we conducted a meta-analysis of secondary outcome. In this analysis, no heterogeneity was found among the included four studies (*I*^2^ value, 0%), so we used a fixed effect model which showed the pooled OR was 0.94 (95% CI, 0.91–0.97; *P* = 0.0005), indicating a protective effect of preadmission intake of statin against the use of advanced nursing practices in critical illness as well (Figure 2B). To further explore the effects of prior statin use, the studies were grouped in the light of outcome definition (30-, 90-day, and hospital mortality). Three studies including 3,900 statin-users and 3,900 non-users reported the effect of preadmission intake of statin on 30-day mortality in critically ill patients (27, 29, 30). Three studies representing 8,873 statin-users and 11,277 non-users reported the effect of preadmission intake of statin on 90-day mortality (28–30). Another three studies including 28,076 statin-users and 28,166 non-users reported the effect of preadmission intake of statin on hospital mortality (26, 30, 31). The *I*^2^ statistic did not change markedly in the subgroup of 30-day mortality (*I*^2^ value, 68%), but it was much lower in the subgroup of hospital mortality (*I*^2^ value, 39%). For studies concerning the outcome of 90-day mortality, the *I*^2^ statistic was 0%. Based on their respective *I*^2^ statistic, corresponding effect models were selected and the subgroup-specific pooled ORs were 0.67 (95% CI, 0.43–1.05; *P* = 0.08) for 30-day mortality, 0.91 (95% CI, 0.83–1.01; *P* = 0.07) for 90-day mortality, and 0.86 (95% CI, 0.83–0.89; *P* < 0.00001) for hospital mortality, respectively (Figures 3A–C).

### Sensitivity Analyses

Because all included studies were observational PSM cohort studies with a low risk of bias (Table 2), a sensitivity analysis based on the methodological criteria was not conducted. In our sensitivity analyses, one study was omitted at a time to evaluate the effect of it on the pooled OR and 95% CI. For the overall mortality outcome, there was no significant change in pooled OR and 95% CI when excluding a single study, suggesting the robustness of this result (Supplemental Figures S1–S6). But the pooled OR and 95% CI of 30-day mortality were significantly different after a single study was omitted (Supplemental Figures S7–S9).

### Evaluation of Publication Bias

To evaluate the publication bias in the included studies concerning the outcome of critically ill patients with preadmission use of statins, funnel plots were used.

For the overall mortality, the funnel plot was asymmetrical at the base as it was missing studies in the bottom left corner suggesting the possibility of publication bias (Supplemental Figure S10). Removal of the Lee study didn't markedly change the asymmetry, implying the potential overstatement of the effect (Supplemental Figure S11). Various degrees of asymmetry was also found in the 30-day and hospital mortality, showing the influence of small sample size (Supplemental Figures S12, S13).

For the 90-day mortality and use of mechanical ventilation, the scatter distribution in funnel plot was roughly symmetrical, implying the absence of publication bias (Supplemental Figures S14, S15).

## Discussion

It is generally believed that the lipid-lowering property of statins make them helpful in preventing progression of cardiovascular diseases, while the pleiotropic properties are suggesting potential utility for improving clinical outcomes for a wide range of diseases (33). Considering the prevalence of systemic inflammatory responses that occur in critically ill patients, we believed that the anti-inflammatory property of statins could be a biologically plausible mechanism of the beneficial effects of statins.

In this meta-analysis, we included six observational PSM cohort studies of 76,766 patients in total and found a potential protective effect of preadmission statin use on mortality in critical illness. For the primary outcome, the pooled OR for all studies showed an overall mortality benefit in patients with critical illness compared with non-users. Hospital mortality and the rate of mechanical ventilation use also demonstrated a significant protective benefit from prior statin use. However, no evidence was found to support that preadmission statin use could provide protective effect for these patients with respect to 30- or 90-day mortality.

Among the included six studies, five of them showed no statistical significance in the primary outcome analysis (27–31), except the one by Ou et al., which accounted for the largest weight of all the studies. Considering the appreciable heterogeneity and possible publication bias in this analysis, we excluded the study by Ou et al. (26), re-analyzed the mortality, and found that there was a marked decrease in the heterogeneity, but the new result still showed a survival advantage of preadmission statin use in critical ill patients. Furthermore, none of the removal of other studies changed the beneficial result, suggesting our result could be considered reliable and robust. The possible source of heterogeneity might be from the Ou study, as its sample was much larger than that of the other five studies, which would lead to unavoidable heterogeneous nature of patients type and statin type. We included the 1-year mortality from this study for primary outcome analysis, a long-term indicator distinct from any other short-term measurements included for the pooled analysis, which might also be a source of heterogeneity.

For the 30-day mortality analysis, there was a trend toward a benefit from prior statin use but the result didn't reach statistical significance, with moderate heterogeneity among the studies. After removal of the studies by Wiewel et al. (30) and Vaduganathan et al. (27), the results didn't substantially change. As the study by Lee et al. (29) was the only one that didn't show a positive effect of statin use on the pooled analysis of 30-day mortality and it had the most weight, we wondered whether the removal of this study could make a difference. As expected, removal of the Lee study showed a different result from before, indicating that preadmission statin use might be associated with a reduced 30-day mortality, with a low heterogeneity. This result was consistent with most of the previous studies, implying the potential beneficial effect on short-term mortality in critical illness. But considering the fact that the sample of included studies was not large enough and a substantial heterogeneity did exist, the likely favorable result from this analysis was not so definite, thus it should be interpreted carefully and more researches are needed.

This meta-analysis didn't document a positive association between prior statin use and 90-day mortality. In a previous meta-analysis, the magnitude of association between statin and infection mortality seemed to be stronger for 30-day mortality than 90-day mortality, implying the positive effect of statin may decrease with time (34). Though the heterogeneity from our 90-day mortality analysis was low, only three studies were included (28–30), limiting the generalization of this result. Considering that patients' medication compliance after discharge may not be as good as hospitalization, and no data for subgroup analysis of longer mortality was presented in this meta-analysis due to the limited number of included studies, the association of prior statin use with 90-day or even longer time mortality remains to be further explored.

Previous meta-analyses have shown an inconsistent association between statin use and mortality in patients with critical illness. An important reason for this discrepancy is the study design. The pooled OR among observational studies generally demonstrated that statin users presented better outcomes compared with non-users (10, 34–36). But these results were challenged to be possibly influenced much by unknown or unquantified confounders. Wan et al. also showed the use of statins was associated with a survival advantage in the meta-analysis from observational studies, but the findings from their meta-analysis of five RCTs demonstrated that there was no significantly positive effect from statin therapy in infection and sepsis (16). This was consistent with the result from the analysis of clinical trials in the study by Ma et al. (34), as well as a later meta-analysis by Deshpande et al. (37). To the best of our knowledge, there was only one RCT showing that statin use was correlated with a lower 28-day mortality (19). However, this benefit was based on a prior statin intake for at least 2 weeks, and *de novo* statin use didn't show the same effect. Besides, no significant difference in 3-month mortality was found in this study. This is similar with our result. For patients with a history of statin prescription, Ou et al. found continued use during hospitalization had a greater reduction in mortality than those who discontinued (26). This possible time/dosage-dependency effect was also reported by other studies (20, 38). Considering the potential pharmacodynamics impairment of stain caused by critical illness (39, 40), enough therapy time seems to be necessary for statin to take the anti-inflammatory effect, and acute statin administration may weaken this protective benefit. However, the “MIRACL” study (41) showed a conflicting result with this, which showed benefits of acute statin use (atorvastatin initiated 24–96 h after presentation with unstable angina or non-Q-wave acute MI) in ACS, decreasing mortality and the occurrence of early, recurrent ischemic events. But it is important to note that only atorvastatin was administrated in that study, and the treatment dose was fixed to 80 mg/day. From the results of the Oh and Ou study, we found that both statin dosage and type might affect the outcomes. Therefore, results might change if the treatment program was performed with different statin type or dosage. In addition, at the time of MIRACL study, different assay methods were used for the diagnosis of an acute coronary syndrome in the participating countries. As the sensitivity of each assay to the identification of MI varied, the diagnosis of MI might have been underestimated and the beneficial effect of statin might be overestimated (42). We believe that further studies concerning the effect of acute statin administration on the outcomes of both acute and chronic critical illness are needed to draw a more explicit conclusion in this issue.

Our meta-analysis has a few strengths. Though all the analyzed studies were observational, we selectively included those that had conducted PSM, which was a robust statistical method to minimize confounders. In addition, the spectrum of disease in the study is broader than previous ones, so our result can be considered reliable and representative.

This meta-analysis has several limitations. First, only three electronic databases and published articles were included for analysis, which could lead to almost unavoidable publication bias, as was shown in funnel plot. But it is difficult to make sure whether the evaluation of publication bias is precise, since the result was obtained only via visual examination and no Egger's regression test was conducted. We have to acknowledge that the result got by funnel plot is generally considered unreliable when the number of the included studies is <10, especially when the heterogeneity is substantial. The method of judging risk of bias is another point which needs to be improved. Though NOS has been used in many meta-analyses, it was criticized heavily due to its unknown validity and its limitation in case-control and cohort studies. Cohort studies are designed to try to assemble a representative exposed cohort, while they frequently fall into the low baseline response, leading to a questionable generalizability of the study. However, unrepresentative exposed cohorts may have a higher baseline response, better exposure assessment, and better follow-up response of cohort members that may result in a higher internal validity of the study findings compared to a cohort study with a representative exposed cohort (43). It is a pity that we didn't use a more comprehensive method like ROBINS-I due to limitation of some objective conditions. Second, despite of the efforts we've made to cover a search field as comprehensive as possible, only six studies were eligible for our inclusion criteria. Besides, there were no RCTs included in this meta-analysis. Although RCTs are required to assess whether a causal relationship exists between statin use and outcomes, we should bear in mind that the widespread use of statins in the primary or secondary prevention of cardiovascular disease would make recruitment of non-users of statins in future prospective studies with adequate power challenging or realistically impossible. Therefore, it may be more practical to choose a scientific and effective approach. In this meta-analysis, we selected PSM observational cohort studies in case of uncontrollable confounding variables. But PSM has its own limitations. In our included studies, the measured confounders didn't overlap, and unbalanced baseline conditions did remain in specific propensity-matched cohort. The unknown confounding variables could lead to bias, such as healthy-user bias in the survival benefit of statin and the surveillance bias. For patients prescribed with statins, they are typically considered to have higher socioeconomic status and health awareness, thus are less likely to develop serious complications. Although proxies for personal economic status or lifestyles were taken into account, not all the included studies controlled such variables as sufficiently as possible. Higher frequency healthcare facility use could lead to higher diagnostic rate and lower severity of diseases, which may give rise to surveillance bias. PSM reduced overall population under study, as all the non-paired patients were removed from analysis. Therefore, the conclusion must be interpreted with caution, and more high-quality studies are needed for confirmation. Third, although the sample size of this meta-analysis was large (76,766 patients in total), at least 80% (62,622 patients) of the study population was diagnosed with sepsis (6,650 patients from the Lee study, 388 patients from the Wiewel study, and 55,584 patients from the Ou study. Data from the Oh study was excluded since it was extracted from a mixed set of diseases, though there was high possibility that sepsis was included in these diseases). Previous meta-analysis that focused on acute lung injury/ARDS reported no mortality benefit of statin therapy in patients (44, 45). In one of our included articles, Wiewel et al. (30) demonstrated that statin use didn't modify systemic inflammatory activation of the vascular endothelium or the coagulation system. This conclusion was of great concern to us because it was inconsistent with the vast majority of current study findings. Taking all these facts into consideration, the effect of statin on outcomes of a broader spectrum of critically ill diseases remain to be studied further. The dominant weight of a single study in the pooled analysis was another limitation, as was in the 30-day mortality analysis, with a significant change of the pooled OR after exclusion of one study. Finally, restricted to the incomplete data of patients, we didn't conduct subgroup analyses by patients' characteristics, types or potency of statins and severity of the illness, though previous studies draw inconsistent conclusions on the effect of statin in terms of these characteristics (46–50).

## Conclusions

We conducted this meta-analysis to describe the association of preadmission statin use and mortality in critically ill patients. The results showed that preadmission statin use is associated with a lower risk of death, and the beneficial effect seems to be short-term. The use of mechanical ventilation is also less in preadmission statin users compared with non-users with an improvement in hospital survival. Further high-quality original studies or more scientific methods are needed to draw a definitive conclusion.

## Data Availability Statement

The raw data supporting the conclusions of this article will be made available by the authors, without undue reservation.

## Author Contributions

S-sY, JJ, G-sW, and YS conceived the meta-analysis. S-sY, JJ, and R-qY extracted all data. B-lW and L-yH undertook and refined the searches. S-sY, G-sW, and YS co-wrote the paper. S-sY and JJ undertook the statistical analyses. All authors contributed to the article and approved the submitted version.

## Conflict of Interest

The authors declare that the research was conducted in the absence of any commercial or financial relationships that could be construed as a potential conflict of interest.

## Abbreviations

PSM: propensity score matching
NOS: Newcastle-Ottawa scale
OR: odds ratio
CI: confidence intervals
RCT: randomized controlled trial
HMG-CoA: 3-hydroxy-3-methylglutaryl coenzyme A
ICU: intensive care units
PRISMA: preferred reporting items for systematic reviews and meta-analysis
ARDS: acute respiratory distress syndrome
MI: myocardial infarction
MI: myocardial infarction
ACS: acute coronary syndromes
MIRACL: myocardial ischemia reduction with aggressive cholesterol lowering.
